# Improving Children’s Behavior in Seven Sessions: A Randomized Controlled Trial of Parent-Child Care (PC-CARE) for Children Aged 2–10 Years

**DOI:** 10.1007/s10578-022-01406-8

**Published:** 2022-08-11

**Authors:** Brandi N. Hawk, Susan G. Timmer, Lindsay A. F. Armendariz, Deanna K. Boys, Anthony J. Urquiza, Erik Fernández y Garcia

**Affiliations:** University of California, Davis Children’s Hospital, 3671 Business Dr., Ste 110, 95820 Sacramento, CA USA

**Keywords:** PC-CARE, Parent-child intervention, Externalizing behaviors, Dyadic intervention, Brief intervention

## Abstract

Parent-Child Care (PC-CARE) is a brief intervention for children with externalizing behaviors designed to address issues with their access to and retention in treatment. A growing evidence base of open trials and comparison studies support PC-CARE’s benefits, but no randomized controlled trials (RCTs) of its effectiveness exist. The current study presents the first RCT of PC-CARE, a 7-session dyadic parenting intervention (trial number removed for blind review). Participants included a racially/ethnically diverse sample of 49 children (29% female) aged 2–10 years and their caregivers. Participants were randomly assigned to PC-CARE or waitlist control. Families participating in PC-CARE showed greater reductions in children’s externalizing behaviors, improvements in children’s adaptive skills, declines in parental stress, and increases in parents’ positive communication skills, compared to families on the waitlist. The results of this first RCT of PC-CARE support the effectiveness of this brief intervention in improving children’s behaviors.

Prevalence rates for externalizing behaviors (e.g., aggression, oppositionality, poor impulse control) are as high as 15–30% among 2-10-year-old children [1, 2]. These behaviors show stability over time and are associated with negative outcomes such as school expulsion and further mental health concerns [2, 3]. Thus, the 2-10-year age range is a critical period for treating externalizing problems, and parenting interventions tend to be recommended, as they have been found to be highly effective for younger children with externalizing problems [4]. Despite the prevalence of externalizing behaviors, the opportune time for intervention, and the fact that effective interventions exist, only 25–50% of these children receive mental health treatment [5, 6].

Many of these children remain untreated due to issues with access to and participation in mental health services, particularly inadequate provider capacity [7]. Among those who do begin services, high attrition rates suggest that keeping families engaged is one of the greatest challenges to providing effective treatment [5, 8, 9]. Thus, researchers suggest an important strategy to increase access and engagement and to maximize treatment effectiveness is to develop briefer interventions that are less time intensive for families and able to be provided in a variety of settings [10]. Parent-Child Care (PC-CARE [11]) is a brief intervention developed to address this goal of increasing access and participation in mental health services for families of 2-10-year-old children with externalizing behaviors.

However, developing briefer interventions, such as PC-CARE, is only the first step. These interventions must be proven to be effective using rigorous scientific methods, thereby establishing their evidence base for this age group. Once the interventions are established as effective, they can be disseminated, implemented, and billed by individuals working with families. A randomized controlled trial (RCT) is the gold standard for proving an intervention’s effectiveness. Although open trials of PC-CARE have supported its benefits [11, 12], to date there have been no RCTs to formally assess its effectiveness. The current study is the first RCT of PC-CARE, offering an important addition to the evidence base on PC-CARE.

## PC-CARE

PC-CARE is a 7-session (1 pre-treatment + 6 treatment sessions) dyadic intervention designed for children aged 2–10 years with mild to moderate externalizing behavior problems. The primary goals of PC-CARE are to improve children’s behaviors (decrease externalizing behaviors; increase positive, adaptive behaviors) and to reduce stress and difficulties within the caregiver-child relationship. The goals are accomplished through teaching and live coaching of caregivers and children to use positive communication strategies, self- and co-regulation strategies, and behavior management strategies.

PC-CARE was founded on research on effective mechanisms in parenting interventions [4, 13, 14], as well as research on parent coaching [15] and implementation in community mental health settings [16]. Some of the most effective evidence-based behavioral parenting interventions involve teaching new skills each week in a 12-week group format (Incredible Years [17]), teaching caregivers skills then using live “bug-in-the-ear” coaching over 14–20 weeks to help caregivers develop “mastery” of those skills (Parent-Child Interaction Therapy; PCIT [18]), teaching new strategies and active skills training weekly for 10 individual or 5 group sessions (Level 4 Triple-P [19]), or using a combination of teaching and video review over 10 sessions (Attachment & Biobehavioral Catch-Up [20]). The primary purpose of developing PC-CARE was to incorporate effective aspects of these interventions into a briefer intervention that would thus address the problems with engagement and attrition due to intervention length as reported in much of the research on longer parenting interventions [5]. Specific process components adapted from these interventions include teaching new skills weekly, assigning weekly homework, and live coaching, found to increase the effectiveness of parenting interventions [13]. Process components that differ from these interventions include actively involving the child in all components of the intervention (e.g., teaching the child to use the skills), live coaching in-room without audio/visual equipment, and assessing motivation at the beginning and end of each session.

In addition to process components, the specific strategies taught in PC-CARE were influenced by these and other child interventions. Positive communication skills include an adaptation of PCIT’s PRIDE (praise, reflect, imitate, describe, enjoy) and Avoid (questions, commands, criticisms) skills. Unlike PCIT, there is no “mastery” criteria, and the descriptions of some skills are adjusted. For example, based on literature demonstrating that questions do not have a negative effect on parent-child interactions [21], PC-CARE treats reflections and descriptions presented as statements and as questions similarly and teaches caregivers to reduce unnecessary questions rather than eliminate them entirely. Self-regulation skills are compilations of deep breathing, muscle relaxation, mindfulness, and coregulation techniques (e.g., labeling feelings, breathing together, massage) taught in many individual therapies for children. Strategies to manage behavior are similar to those taught in other parenting interventions (e.g., selective attention, modeling, rules, effective commands), with the exception of timeout. Timeout was not included in the PC-CARE protocol due to concerns about being able to help caregivers use timeout effectively in such a short timeframe. Instead, PC-CARE teaches removal of privileges as a consequence for non-compliance with commands.

### ***Current Evidence Base for PC-CARE***

PC-CARE has been provided to diverse populations of children and caregivers with low attrition rates and improvements in children’s behaviors [11, 12]. In an open-trial of PC-CARE at a university-affiliated outpatient community mental health clinic, services were provided to children with externalizing disorders, autism spectrum disorders, trauma histories, and problematic sexual behaviors, and with caregivers who were biological, foster, or adoptive. Most families had Medicaid, and referrals came primarily from the family (self-referral), child welfare, and pediatric providers. Participation in PC-CARE was related to reductions in externalizing behaviors and parenting stress, as well as increases in parents’ use of positive communication skills from pre- to post-intervention [11]. PC-CARE has also been delivered to foster caregivers of 1-5-year-old children in new foster placements as a secondary-prevention (i.e., offered to all foster families regardless of child’s behaviors). PC-CARE was conducted primarily in the family’s home by licensed, license-eligible (e.g., student, pre-licensure), and non-license eligible (e.g., Bachelor’s degree) providers. Participation in PC-CARE was associated with reductions in externalizing behaviors, improvements in adaptive skills (e.g., initiative to meet needs, self-regulation), and increases in caregivers’ use of positive communication skills [12]. Children who completed PC-CARE also showed greater placement stability than those who did not [12].

PC-CARE outcomes have also been compared to those of PCIT in families from the same mental health clinic, though the study was not randomized. Primary differences between PCIT and PC-CARE include length, skills taught, inclusion of child, “mastery” criteria, and equipment needed. PCIT typically lasts 14–20 sessions compared to PC-CARE’s 7. PCIT is divided into two components that emphasize different skills (Child Directed: PRIDE, avoids, selective attention; Parent Directed: direct commands, timeout), whereas PC-CARE teaches a total of 22 skills and teaches new skills each week. PCIT teaches caregivers skills without the child, while PC-CARE actively teaches the child and caregiver together. PCIT required the caregiver to reach a “mastery” of the skills, compared to no “mastery” criteria in PC-CARE. Finally, PCIT uses a two-way mirror and bug-in-the-ear technology to coach, whereas PC-CARE coaches can use that equipment or remain in the room with no equipment. The study assessed treatment retention and behavior change during the seven weeks of PC-CARE and the first half of PCIT (Child Directed, roughly seven weeks). Children who participated in PC-CARE had higher treatment retention and showed greater improvements in child externalizing behaviors during that time period than PCIT participants [22].

## Current study

### Purpose of study

Expanding the evidence base for briefer interventions for children with externalizing behaviors is an important step toward increasing provider capacity and family engagement [9]. Part of this process is establishing interventions as evidence-based, disseminating, and implementing them. The purpose of the current study was to add to the evidence base for PC-CARE by conducting the first RCT to test the effectiveness of providing PC-CARE to 2-10-year-old children with externalizing behaviors compared with a waitlist control. As no RCTs of PC-CARE exist to date, this study is necessary for establishing PC-CARE as an evidence-based intervention.

### Hypotheses

We hypothesized that caregivers who completed PC-CARE would report greater reductions in externalizing behaviors and improvement in adaptive skills for their children, report less parenting stress for themselves, and be observed to use more positive communication skills than caregivers of children in the waitlist control.

## Methods

### Participants

#### Inclusion/Exclusion Criteria

Study inclusion criteria included: (1) child was 2.00 to 10.99 years old, (2) child had one participating primary caregiver who lived with the child at least 50% time, (3) child was a pediatric patient within a university health system, (4) family was able to participate in services in English, and (5) caregiver reported child had challenging behaviors. There were no inclusion or exclusion criteria related to diagnosis or minimum behavioral score for participation.

#### Sample

Study participants were referred from two pediatric clinics. At clinic 1, children were diverse, representative of the general population in the area: 45% were Caucasian, 19% African American, 17% were Asian, 19% were Latinx and other races and ethnicities. Children ranged in age from infancy to over 21 years: 31% were under 3 years, 17% were 3–5 years, 20.5% were 6–10 years, and the remaining 31.5% were 11 years and older; 56% of children were male. The clinic saw insured and uninsured children, though the large majority were insured: 62% had PPO/HMO, 31% were insured by Medicaid, 4% were covered by CHAMPUS (military insurance), and 3% were uninsured. At clinic 2, children were 61% Caucasian, 10% African American, 20% Asian, 19% Latinx and other races and ethnicities. Children ranged in age from infancy to over 21 years: 31% were under 3 years, 17% were 3–5 years, 20.5% were 6–10 years, and the remaining 31.5% were 11 years and older; 56% of children were male. The clinic saw insured children only: 97% had PPO/HMO, 2.5% were covered by CHAMPUS (military insurance), and 0.5% were insured by Medicaid.

**Fig. 1:**
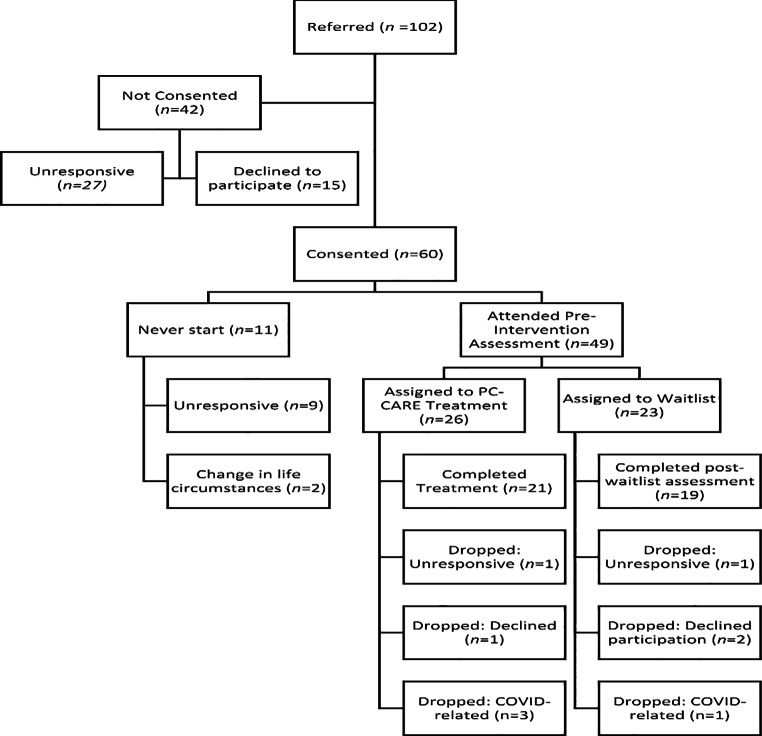
*Flow diagram for referral and participation in the PC-CARE study*

Primary care pediatricians from these clinics referred 102 eligible children to this study from September 2018 to March 2020. Of these, 27 families did not respond to communication attempts from the study team, and 15 families declined participation. Parents of 60 eligible children consented to participate in the study. Eleven families were not randomized, either because they did not respond to therapist calls to schedule (*n* = 9) or they declined participation due to external circumstances (i.e., birth of a child, change in work schedule; *n* = 2), leaving a total sample of 49 families who consented to participate, attended an initial assessment, and were randomized to intervention group (see Fig. 1).

Children in the current sample were representative of those seen in the two clinics. Children ranged in age from 2 to 7 months to 10 years 6 months (*M* = 5.35; *SD* = 2.04), and ethnicities included African American (18%), Latinx (18%), Asian American/Middle Eastern/Pacific Islander (14%), and Caucasian (49%). Unlike the clinic populations, only 14 children (29%) in the study were female (see Table 1).

**Table 1:** *Descriptive differences between families in the treatment and waitlist groups*

	Treatment (N = 26)	Waitlist (N = 23)	Effects
Child’s age	5.22 (2.08)	5.47 (2.04)	*F*(1, 47) = 0.19, *p* = .67
Child’s sex (% Male)	73.1%	69.6%	*χ*^*2*^ = 0.07, df = 1, 49, *p* = .79
Child’s ethnicity % African American % Caucasian % Latinx % Other ethnicity	11.5 53.8 23.1 11.5	26.1 43.5 13.0 17.4	*χ*^*2*^ = 2.64, df = 3, 49, *p* = .45
Relationship of caregiver: % Biological parent % Adoptive/legal guardian % Foster parent	92.3 7.7 0.0	91.3 4.3 4.3	*χ*^*2*^ = 1.36, df = 2, 49, *p* = .51
Caregiver’s ethnicity % African American % Caucasian % Latinx % Other race/ethnicity	9.1 59.1 18.2 13.7	29.4 47.1 5.9 17.6	*χ*^*2*^ = 3.70, df = 3, 39, *p* = .30
Caregiver Education % Some High School % High School % Some College % College Graduate % Post-Secondary	4.0 4.0 16.0 24.0 52.0	0.0 0.0 39.2 39.2 21.7	*χ*^*2*^ = 8.01, df = 4, 48, *p* = .09

Caregivers were predominantly female (86%) and included 45 (92%) biological parents, 3 (6%) adoptive or legal guardians, and 1 (2%) non-relative foster caregiver. They identified racially/ethnically as African American (14%), Latinx (10%), Asian America/Middle Eastern/Pacific Islander (12%), and Caucasian (43%); 21% chose not to identify their ethnicity. One parent had not finished high school (2%), one (2%) had completed high school, 27% attended some college, 31% had college degrees, and 38% had a post-secondary education (see Table 1).

#### Therapists

Three therapists provided services for this study: two licensed psychologists and one licensed clinical social worker. Two were community providers working in a community mental health clinic within an academic medical center; one was a retired volunteer. All were trained to PC-CARE certification (i.e., demonstrate 25 competencies during live sessions & graduate two cases) prior to working on this study. Therapists attended monthly meetings with other PC-CARE providers to practice observational coding and consult on cases, as well as weekly supervision with the principal investigator (PC-CARE co-developer).

### Procedures

The [removed for blind review] institutional review board approved and monitored all facets of this study. This trial was registered with clinicaltrials.gov (removed for blind review). This study was of minimal risk to participants and not intrusive. There was some threat to privacy, which was overcome by assigning subject IDs and keeping names in a separate, password-protected table. Due to ethical concerns around waitlist participants not receiving needed intervention, all waitlist participants were offered PC-CARE after completing the waitlist. Informed consent was obtained from all individual participants included in the study.

#### Referral process

Thirty primary care physicians, a mix of trainee (residents), faculty, and non-academic practitioners, at two urban university pediatric clinics in [state removed for blind review] introduced caregivers to the study at pediatric visits if the caregivers mentioned that they were concerned about a child’s behavior, concerned about their own ability to parent those behaviors, or the pediatric provider observed behaviors which would possibly qualify for PC-CARE. Before starting recruitment, the investigators met with pediatricians to explain the PC-CARE program, discuss observed behaviors or parent concerns that would warrant referral to the program, and teach physicians how to make referrals.

We requested that pediatricians continue to refer patients to mental health treatments as they normally would for a given case presentation and not to stop any current treatments. In other words, participation in the PC-CARE study was not replacing usual clinical care, and children in the waitlist control received treatment as usual pending the start of their intervention. We took this approach purposefully because as usual care in these clinics, pediatricians would routinely recommend several simultaneous interventions for such behaviors, including but not limited to behavioral health referrals, online resources, workbooks, etc. Therefore, referral to PC-CARE in addition would be in keeping with usual practices. Despite these instructions, and consistent with known limited access to pediatric mental health services, only one child in this study was connected to another mental health provider at the start of PC-CARE. This same child was unable to complete the treatment protocol due to COVID-19 (see below) and is not included in outcomes.

Physicians referred children directly to the study team, providing only child name, caregiver name, and caregiver contact information. Physicians asked parents if the study team could contact them to explain the study and gave study fliers to parents at the time of referral. The fliers contained simple information describing PC-CARE, the research program, and the possibility of being assigned to a waitlist control condition. Pediatricians were not asked to keep track of how many parents refused pediatricians’ offers to be contacted by the study team. After receiving the referral, a research assistant (RA) made up to four attempts to contact the family via phone or email to explain the study in more detail and to obtain research and video consent prior to beginning the assessment. After the parent provided research consent, a study therapist made up to four attempts to contact them to conduct a phone intake interview and to schedule a pre-intervention assessment. The phone intake interview included questions about the family, presenting problems, and medical, social, and educational background. Child assent was obtained in-person prior to beginning the intake assessment for children six years and older. Families were compensated with a game worth approximately $15 after completing the post-treatment assessments and the post-waitlist assessments.

#### Intake Assessment

All components of the intake assessment occurred prior to randomization; thus, the RA, families, and therapists were blind to group allocation during the assessment. The RA emailed links to a secure online portal containing behavioral questionnaires, which were to be completed prior to the assessment appointment. At the appointment, parents completed additional paper-and-pencil behavioral questionnaires and participated in a video-recorded 12-minute observational assessment of the parent-child dyad as they played together in three semi-structured play situations that are analogs of typical parent-child interactions. Each of the 4-minute scenarios (i.e., child-led play, parent-led play, clean up task) were coded in the moment by the therapist for clinical purposes. After the observation, the family was assigned to the treatment or waitlist group per the randomized allocation protocol. If the family was assigned to the treatment group, the therapist provided additional information about PC-CARE, presented information about why children display difficult behaviors (e.g., trauma, autism spectrum disorder, temperament), and identified treatment goals. If the family was assigned to the waitlist group, the therapist provided additional information about when they would be contacted to return for treatment.

#### Randomization

Participants were randomized to treatment or waitlist groups using a block urn design [23], which provides high allocation randomness while providing imbalance control, ensuring study therapists were consistently providing PC-CARE to both treatment and post-waitlist participants. A volunteer unaffiliated with the study placed the group allocations in sealed envelopes numbered sequentially, leaving all study personnel blind to participants’ group allocation until the initial assessment was complete. The research therapist opened the envelopes sequentially.

#### Intervention

PC-CARE treatment consisted of 6 weekly, 50-minute treatment sessions. Children and caregivers participated together for the entirety of sessions. Treatment sessions consisted of the following components: (1) Caregivers completed the WACB-N [24], a 9-item measure of child behavior, and therapists conducted a brief check-in about the week with caregivers and children. (2) Therapists taught positive communication, calming, and/or behavior management skills to caregivers and children during a 10-minute didactic. New skills were taught each week and built upon each other (see Table 2). Therapists taught caregivers how to use these skills with their children and often taught children how to use the skills with their caregivers (e.g., praise, requesting transitions) and/or siblings and peers (e.g., selective attention, choices). (3) Therapists conducted a 4-minute observation of child and caregiver playing together, coding caregivers’ use of five specific positive communication skills (praise, reflect, imitate, describe, enjoy; PRIDE), any strategies used to manage behaviors, and qualitative aspects of the interaction. (4) Therapists coached caregivers to use the skills within the context of play, pointing out the impact of the skills on children, for 20 min. Throughout coaching, therapists also occasionally instructed children to use the skills with their caregivers, pointing out the caregivers’ response. The frequency of coaching the child varied based on the child’s developmental level. Therapists used a combination of leading (e.g., “tell your child how much fun you’re having”), following (e.g., “you’re doing a great job modeling”), providing brief psychoeducation (e.g., “those choices offer your child a sense of control”), observing children’s responses (e.g., “when you practiced deep breathing, your child followed along”), and generalizing skills to other settings (e.g., “you can use that same praise when your child sits down for meals”) in their coaching. (5) Therapists reviewed accomplishments made by the caregiver and child in session and assigned “Daily CARE”, which involved the caregiver and child spending 5 min in play daily, as well as using skills throughout the day. One week before the 6th treatment session, research assistants emailed links to the secure online portal containing behavioral questionnaires, which were to be completed prior to the final session. During the 6th treatment session, caregivers completed additional post-treatment paper-and-pencil behavioral measures and participated in a 12-minute observation with the child. Therapists then reviewed all the skills taught during PC-CARE with the caregivers and children and helped the family prepare for future difficult behaviors. Therapists contacted all families one-month after treatment ended to obtain a WACB-N and offer a booster session if the family wanted one. An RA contacted the family six-months after treatment, administering a WACB-N.

**Table 2:** *Strategies Taught in PC-CARE by Session*

Session Number	Strategies Taught
1	*Positive Communication*: Praise behaviors, Reflect words, Imitation actions, Describe behaviors, Enjoy time together, Avoid negatively phrased comments, Avoid criticisms, Reduce questions and commands *Strategies to Manage Behavior*: Transitions, Compliance Friendly Environments
2	*Strategies to Manage Behavior*: Selective Attention, Redirection, Modeling *Regulation*: Calming/coping skills; Coregulation skills
3	*Strategies to Manage Behavior*: Rules, Choices, When-Then/If-Then statements
4	*Strategies to Manage Behavior*: Effective Commands, Consistent Consequences (praise for compliance; removal of privilege for non-compliance)
5	*Strategies to Manage Behavior*: Redoing *Regulation*: Recovery after commands and/or difficult behaviors
6	Review all strategies; identify which work best for the family

PC-CARE also incorporates multiple tools intended to increase engagement and motivation. At the end of the pre-treatment session, families are asked five questions assessing their belief about the treatment’s effectiveness and their commitment to engage in the services as the start of a lifestyle change. Each week, families are shown a graph of their weekly progress (i.e., child WACB scores, caregiver PRIDE skills, days of Daily CARE) to review gains and goals. At the beginning of session, therapists ask how well the previous week’s strategies worked over the past week on a 1 (not at all well) -5 (extremely well) Likert scale; at the end of session, therapists ask how well the family thinks that day’s strategies will work for them (also on a 1–5 Likert scale). Therapists use techniques consistent with motivational interviewing when conducting these interviews (e.g., what would need to happen to change this score to a 4?).

#### Waitlist Control

Families placed on the waitlist received no PC-CARE services and were contacted after six weeks (the length of PC-CARE) to attend another assessment and begin treatment. The second assessment included the online and in-person administered behavioral measures, a 12-minute parent-child observation, and an interview to obtain updates on the child’s functioning. Therapists also provided a description of treatment, information about why the child may display difficult behaviors (e.g., trauma, autism spectrum disorder, temperament), and worked with the family to identify treatment goals. Families on the waitlist then received the full PC-CARE intervention, including one-month and six-month follow-ups.

#### Treatment location

Study therapists provided services in either the pediatric primary care clinic (*n* = 29) or an outpatient mental health clinic (*n* = 19) associated with the same university health system. The decision to provide services in one site or the other was dictated by family schedule and preference. The primary care office had a desk and small floor space. During coaching, dyads either sat next to each other at the desk with the therapist seated across from them or on the floor with the therapist at the desk. The outpatient mental health clinic room was larger, with a table and large floor space. During coaching, dyads either sat next to each other at the table with the therapist seated across from them or on the floor with therapist slightly behind the caregiver on the floor or seated on a chair removed from the dyad.

#### Impact of COVID-19

This study took place from September 2018 to July 2020. Stay at home orders related to the COVID-19 pandemic took effect at the end of recruitment in mid-March 2020. At that time, two participants from the treatment group were in the middle of treatment (sessions 2 and 3), one participant from the waitlist group was supposed to return for a post-waitlist assessment, and two participants from the waitlist group had just completed their pre-intervention assessment. The two treatment and one post-waitlist participant ended participation at this time, despite multiple attempts to contact them and offer to return to services once stay at home orders were lifted. The two waitlist participants, along with three new participants (2 treatment, 1 waitlist), participated according to study protocols once stay at home orders were modified for medical settings in May 2020. The only protocol modification was that participants and therapists wore surgical masks during sessions. One additional treatment participant had significant medical concerns, placing him in the high-risk category for infection. After attending an initial assessment, this participant was unable to go to the clinic to participate in sessions, which made them ineligible for the study. The family received consultation with a therapist to meet their mental health needs but was counted as having discontinued the study.

### Measures

#### The Behavior Assessment System for Children, 3rd Edition (BASC-3)

The BASC-3 [25] is a comprehensive and standardized measure of child behaviors, encompassing both clinical behaviors and adaptive skills. The parent rating scales (PRS) for preschool (2–5 years; 139 items) and child (6–11 years; 175 items) were used in this study. The BASC-3 PRS provides four indices (Behavioral Symptoms, Externalizing Problems, Internalizing Problems, Adaptive Skills) and 12 (preschool) or 14 (child) subscales. According to the clinical manual, *T* scores of 60–69 are considered ‘at risk’ and scores of 70 and above are considered ‘clinically significant’ for the clinical scales; *T* scores of 31–40 are considered ‘at risk’ and scores of 30 and below are considered ‘clinically significant’ for the adaptive scales. The BASC-3 has demonstrated good reliability and validity, with Cronbach’s alpha scores ranging from 0.77 to 0.93 and construct validity demonstrated with moderate to high correlations with other clinical and adaptive scales [25]. The current study used the Externalizing Problems and Adaptive Skills Indices. Cronbach’s alphas in the current dataset are as follows: Preschool Externalizing Problems α = 0.85, Preschool Adaptive Skills α = 0.77, Child Externalizing Problems α = 0.91, Child Adaptive Skills α = 0.91.

#### Parenting stress index, 4th Ed. – short form (PSI4-SF)

The PSI4-SF [26] is a 36-item questionnaire that assesses three sources of parenting-related stress: stress related to the parenting role (Parental Distress), stress related to perceived problems in the parent-child relationship (Parent-Child Dysfunctional Relationship), and stress related to the child’s difficult behaviors or temperament (Difficult Child). A Total Stress scale reflects the sum of the three scales. *T* scores are created for the three subscales and Total Stress scale. The PSI4-SF has demonstrated validity and reliability, with Cronbach’s alpha coefficients α > 0.80 for each scale. The current study used the Total Stress scale, which had a Cronbach’s alpha coefficient α = 0.90 in the current sample.

#### PC-CARE coding system

Parents and children participated in a structured 12-minute observation at pre- and post-intervention. The observation included three 4-minute periods: a child-directed interaction in which parents were instructed to follow along with their child’s play, a parent-directed interaction in which parents were instructed to get the child to play according to the parents’ rules, and a clean-up task in which parents were instructed to have the child clean up by themselves. Parent verbalizations and behaviors were coded during the 4-minute child-directed interaction using the PC-CARE Coding System [27], a microanalytic behavioral coding system used to code caregiver verbalizations and behaviors and adapted from the Dyadic Parent-Child Interaction Coding System (DPICS, 4th Ed. [28]). Although many similarities exist between the PC-CARE Coding System and DPICS, the criteria for specific codes are broader and the coding priority order is different in the PC-CARE Coding System. A total of 19 different codes distinguishes among five different kinds of verbalizations (e.g., PRIDE skills, questions, commands) and 14 behaviors (e.g., modeling, redirecting). In this study, we focused on the parent’s use of verbalizations associated with positive parenting communication (i.e., PRIDE skills). PRIDE skills included the following statements:

- Praise- A positive evaluation of the child, including both nonspecific (e.g., “Nice!”), and specific praise (e.g., “Nice work playing gently with the toys!”).
- Reflections- Repetition or rephrasing the child’s appropriate verbalizations (e.g., Child: “I’m building a house.” Parent: “You are building a house.” Child: “Bwoo flower!” Parent: “A blue flower?”).
- Imitation- An overt statement indicating that the caregiver is following the child’s lead (e.g., Parent: “I’m driving my car just like you.”).
- Behavioral Descriptions- A non-evaluative description of the child’s behavior (e.g., “You are drawing a rainbow.”) or progress toward goals (e.g., “You are really concentrating.”).
- Enjoyment- A verbal expression of positive feelings about the current situation that would not be considered praise (e.g., “I’m having fun playing with you.”).

Therapists received approximately 3 h of coding training and needed to code 10 times with 80% reliability with their trainer to be considered “competent.” Once reaching coding competence, therapists coded monthly during team meetings to maintain fidelity in coding. Therapists’ codes are used in this study. Research assistants blind to participants’ treatment group and assessment number, who also reached competency standards, recoded approximately 15% of the video recordings used in this study. In a sample of 32 observations, results of intraclass correlational analyses between providers’ codes from live sessions and research assistants’ codes from the session’s video recording for PRIDE skills showed reliability at alpha = 0.89, demonstrating good reliability.

### Fidelity

All sessions were video recorded. Videos were randomly selected (no more than one from each dyad; N = 39) and reviewed for fidelity. We measured the amount of time spent conducting the didactic, whether the specified didactic topics were provided, and the amount of time spent coaching. To ensure reliability among those evaluating fidelity, research assistants received training in how to measure sessions’ fidelity factors. We recoded 8 of the sessions that had been coded for fidelity to estimate the reliability of fidelity coders. Intraclass correlation coefficients of time measurements were all at least *r* = .97 or higher, and binomial measures showed between 88 and 100% agreement on codes of the presence vs. absence of different didactic topics, suggesting high coding reliability. The results of analyses of fidelity showed that each sessions’ topics were highly likely (100%) to have been covered in that session, that the amount of time spent in the “10-minute” didactics was acceptable, averaging 7.51 min (SD = 2.27) and ranging from 1.46 to 12.47 min. The average time spent coaching (target time 15–20 min) at each session ranged from 10 to 25 min, with an average of 18.00 min (SD = 3.51).

### Analysis strategy

Preliminary analyses checked the randomization of groups and potential effects of attrition. Primary analyses used Repeated Measures Analysis of Variance (RM-ANOVA), with assessment point (pre- and post-intervention) as the within-subjects variable and group (treatment vs. waitlist) as the between-subjects variable, to assess intervention effects on outcomes of primary interest (i.e., externalizing behaviors, adaptive functioning, total parenting stress, parent positive communication skills). Although families in the waitlist group were offered PC-CARE after waiting, assessment point for primary analyses includes only pre- and post-treatment for the treatment group and pre- and post- waitlist for the waitlist group. Power analyses showed that we should be able to detect a large-sized effect (i.e., f = 0.40, eta-squared = 0.138) with a power of 80 [29], which is appropriate for a wait-list control design since we expect large-sized effects based on outcomes of other RCTs of brief parenting programs [9]. Analyses were conducted using the sample of participants with pre- and post-intervention data for each measure, and again using an intent-to-treat design.

## Results

### Randomization check

There were no significant differences between the treatment (*N* = 26) and waitlist (*N* = 23) groups in child age at enrollment, caregiver age, child sex, child race/ethnicity, caregiver race/ethnicity, caregiver education, caregiver relationship to child, or treatment location (all *p* > .05; see Table 1). The groups also did not differ by length of time from pre- to post-intervention assessment or rate of study dropout. Four participants from the waitlist group (17%) and five participants from the treatment group (19%) did not complete the research protocol (*p* = .87). Families in the waitlist group had a mean of 8.74 weeks (SD = 3.32) between assessments, while the treatment group had a mean of 8.28 weeks (SD = 2.67) between assessments (*p* = .95). Families who did not complete the study did not differ from those who completed the study on any of the demographic or pre-intervention outcome variables (all *p* > .05).

### Primary results

#### Child externalizing behaviors

**Fig. 2:**
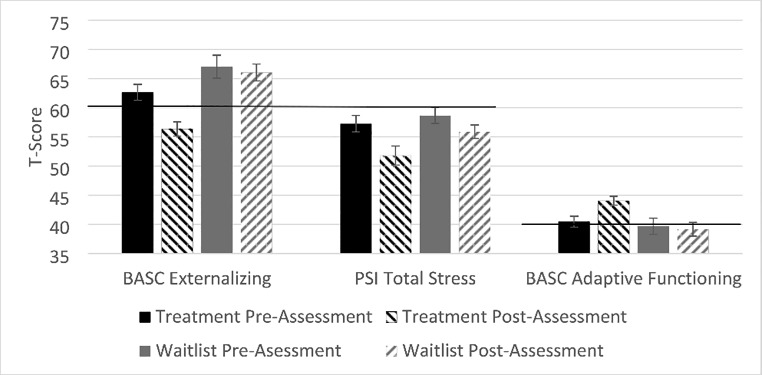
*Mean (standard error of the mean) T-score changes from pre- to post-intervention for families in the PC-CARE treatment group and waitlist group. For the BASC Externalizing and PSI Total Stress scales, higher scores indicate worse problems, with scores above T = 60 indicating clinical concern. On the BASC Adaptive Functioning scale, lower scores indicate worse functioning, with scores of T = 40 and below indicating clinical concern. For each measure, families who completed PC-CARE showed greater pre- to post-intervention improvements than families in the waitlist group*

As shown in Table 3 and illustrated in Fig. 2, caregivers who participated in PC-CARE reported significantly greater reductions in their children’s externalizing behavior problems than parents in the waitlist group, *F*(1,37) = 6.76, *p* = .01, *η*^*2*^ = 0.15. At pre-intervention, similar percentages of children in the waitlist and treatment groups were reported as having at-risk or clinical levels of externalizing problems (treatment: 65%; waitlist: 63%), *Χ*^*2*^(2, *N* = 39) = 3.24, *p* = .20. However, at post-intervention, only 40% of children in the treatment group were reported as having at-risk levels of externalizing symptoms or greater versus 79% of children in the waitlist group*Χ*^*2*^(2, *N* = 39) = 12.05, *p* = .002.

**Table 3:** *Means (SD) and ANOVA Results of Intervention Effects for Children in the Treatment and Waitlist Groups*

Measure	Treatment		Waitlist		Effects
	Pre-Assess	Post-Assess	Pre-Assess	Post-Assess	
BASC Externalizing Problems (*N*_*T*_=20, *N*_*W*_=19)	62.65 (8.40)	56.35 (7.65)	67.05 (12.26)	66.05 (9.09)	AxG: *F*(1,37) = 6.76, *p* = .01, *η*^*2*^ = 0.15, Power = 0.72 A: *F*(1,37) = 12.82, *p* = .001, *η*^*2*^ = 0.26, Power = 0.94 G: *F*(1,37) = 6.08, *p* = .02, *η*^*2*^ = 0.14, Power = 0.67
BASC Adaptive Functioning (*N*_*T*_=20, *N*_*W*_=19)	40.45 (5.93)	44.05 (4.79)	39.68 (8.62)	39.16 (7.28)	AxG: *F*(1,37) = 6.00, *p* = .02, *η*^*2*^ = 0.14, Power = 0.67 A: *F*(1,37) = 3.33, *p* = .08, *η*^*2*^ = 0.08, Power = 0.43 G: *F*(1,37) = 2.00, *p* = .17, *η*^*2*^ = 0.05, Power = 0.28
PSI Total Stress (*N*_*T*_=20, *N*_*W*_=17)	57.25 (8.67)	51.80 (9.97)	58.66 (8.31)	55.88 (6.99)	AxG: *F*(1,35) = 5.97, *p* = .02, *η*^*2*^ = 0.15, Power = 0.66 A: *F*(1,35) = 12.54, *p* = .001, *η*^*2*^ = 0.26, Power = 0.93 G: *F*(1,35) = 0.48, *p* = .50, *η*^*2*^ = 0.01, Power = 0.10
Coding PRIDE (*N*_*T*_=21, *N*_*W*_=18)	7.38 (5.71)	14.24 (5.80)	7.56 (5.07)	6.61 (5.79)	AxG: *F*(1,37) = 9.70, *p* = .004, *η*^*2*^ = 0.21, Power = 0.86 A: *F*(1,37) = 5.57, *p* = .02, *η*^*2*^ = 0.13, Power = 0.63 G: *F*(1,37) = 8.25, *p* = .007, *η*^*2*^ = 0.18, Power = 0.80

*Note.* N_W_ = sample size for waitlist group; N_T_ = sample size for treatment group; BASC = Behavior Assessment Scale for Children, 3rd Edition; PSI = Parenting Stress Index Short Form, 4th Edition; PRIDE = praise, reflection, imitation, description, and enjoyment verbalized during a four minute child-led play observation; A = Assessment Point main effect; G = Group main effect; AxG = assessment point by group interaction

#### Child adaptive skills

Children who received the PC-CARE intervention were reported to display greater increases in adaptive skills (i.e., social skills, functional communication, leadership, activities of daily living, adaptability) from pre- to post-intervention than children in the waitlist group, *F*(1,37) = 6.00, *p* = .02, *η*^*2*^ = 0.14 (see Table 3; Fig. 2). Prior to the intervention, 40% of children in the treatment group and 58% of children in the waitlist group were described as having at-risk or clinically significant deficits in adaptive functioning, *Χ*^*2*^(2, *N* = 39) = 1.47, *p* = .48. After the intervention, only 15% of children in the treatment group compared to 74% of children in the waitlist group were described as having these deficits, *Χ*^*2*^(2, *N* = 39) = 13.78, *p* = .001.

#### Parenting stress

Caregivers in the treatment group reported significantly larger reductions in parenting-related stress from pre-to post-intervention than caregivers in the waitlist group, *F*(1,35) = 5.97, *p* = .02, *η*^*2*^ = 0.15 (see Table 3; Fig. 2). While the mean stress levels reduced, the percentage of caregivers reporting parenting-related stress levels above the clinical cutoff was low for both the treatment and waitlist groups at both pre-intervention (treatment: 30%, waitlist: 29%), *Χ*^*2*^(1, *N* = 37) = 0.002, *p* = .97, and post-intervention (treatment: 25%, waitlist: 24%), *Χ*^*2*^(1, *N* = 37) = 0.01, *p* = .92.

#### Parenting skills

Caregivers’ positive, child-directed verbalizations (PRIDE) were coded during a 4-minute child-led play observation. As seen in Table 3, caregivers in the treatment group were observed to have greater increases in PRIDE use from pre- to post-intervention than caregivers in the waitlist group, *F*(1,37) = 9.70, *p* = .004, *η*^*2*^ = 0.21. Similarly, the overall proportion of verbalizations during the four minute observation that were coded as PRIDE increased for caregivers in the treatment group (pre-intervention: *M* = 0.16, *SD* = 0.09; post-intervention: *M* = 0.32, *SD* = 0.13) but not for those in the waitlist group (pre-intervention: *M* = 0.16, *SD* = 0.10; post-intervention: *M* = 0.13, *SD* = 0.08), *F*(1, 37) = 17.98, *p* < .001, *η*^*2*^ = 0.33.

### Intent to treat analyses

Intent to treat analyses were conducted using the Last Observation Carried Forward (LOCF) method to impute post-intervention data missing due to attrition [30]. All differences described above remained statistically significant when analyzed using the intent to treat design.

## Discussion

While PC-CARE has been associated with positive child outcomes in two open-trials [11, 12] and a non-random comparison with PCIT [22], the current study is the first RCT of PC-CARE’s effectiveness. Results of this RCT support PC-CARE’s identification as an evidence-based treatment and suggest that PC-CARE is effective in reducing children’s parent-reported externalizing behaviors, improving their adaptive skills, reducing parental stress, and increasing parents’ use of positive communication skills.

As hypothesized, results of this study suggest that in approximately two months, families with 2-10-year-old children participating in PC-CARE are likely to report improved externalizing behavior problems and stress in the parent-child relationship. While other effective interventions for this population exist, they are often time consuming, have high attrition rates, and may not be easily adapted to various intervention sites [9, 10]. PC-CARE was designed to address these concerns by being relatively brief (7 sessions), incorporating strategies to keep families engaged (e.g., teach new skills each week, show weekly progress, incorporate motivation questions, actively include the child at all times), and having few requirements related to treatment room setup or location. The current study confirmed the effectiveness of these strategies. The seven sessions, conducted in multiple settings, were completed in a mean of 8.28 weeks with an 81% retention rate. These findings not only contribute to PC-CARE’s evidence-base as a treatment that can improve outcomes for children with externalizing problems, but also provides evidence that PC-CARE may increase access to services by addressing the attrition, length, and location barriers of other parenting interventions [4, 9].

Additionally, the effect sizes in this study compared favorably to those of other established effective interventions. The effect size we found for externalizing problems (*η*^2^ = 0.15; Cohen’s *d* = 0.84) was consistent with waitlist comparison effects found for PCIT (*d* = 0.61–1.45) and Triple P (*d* = 0.60 − 0.69) in a meta-analysis of the two interventions [31]. The similar effect size of PC-CARE compared to two of the most common and effective, yet more resource intense, parenting interventions supports the promising nature of the relatively briefer intervention which can be implemented in various settings, such as primary care offices.

With as few as 25% of children with externalizing behaviors receiving mental health treatment [6] and high attrition rates for those that do [4], there is a need for effective interventions that can keep families engaged, via being less time intensive and being provided in settings where families already feel comfortable and engaged [9]. The current findings, combined with the comparison study of PCIT and PC-CARE [22], suggest that families can obtain similar treatment gains with a shorter time commitment. If briefer treatments can produce similar outcomes as longer treatments, then not only could families find success more quickly, but treatment providers would be able to work with more families and have shorter waiting lists.

Although the primary behavioral target for PC-CARE is children’s externalizing problems, participation in PC-CARE also resulted in improvements in children’s adaptive skills. While these are related constructs (e.g., when children lack adaptive skills, they may try to get their needs met through externalizing), some parenting interventions have demonstrated improvements in children’s adaptive skills and communication but not externalizing behaviors [32–34]. In contrast, many studies of parenting interventions designed to improve externalizing behaviors do not include adaptive functioning as an outcome unless testing the intervention’s effectiveness with children with autism spectrum disorder [35–37]. Only one study comparing individual and group PCIT found improvements in children’s externalizing problems and adaptive skills with both treatments [38]. In demonstrating its effectiveness in improving children’s adaptive skills, PC-CARE steps forward as a promising practice for supporting positive development in populations with ongoing developmental or medical problems.

Not only did children’s behaviors improve with PC-CARE, but, as hypothesized, caregivers also demonstrated improvements in their positive communication skills and reported less parenting stress after completing treatment. Caregivers who completed PC-CARE doubled their total number and proportion of PRIDE skills from pre- to post-treatment, whereas caregivers in the waitlist group did not. Positive communication skills are foundational for positive caregiver-child relationships [39]. Caregivers’ skill improvement may be a proxy for more positive interactions and relationships, and it confirms that treatment was effective in helping caregivers use the skills that were taught. Parenting stress is often related to child externalizing behaviors [40]. More research is needed to determine causal relationships between skill use, parenting stress, and child externalizing behaviors; however, results of this study suggest that PC-CARE supports caregivers in feeling less stressed in the parenting role and in learning and incorporating positive communication skills in their interactions with their children.

## Study Limitations and directions for Future Research

While the results of this study are promising, there are limitations with using a waitlist control in this RCT. Results indicate that participating in PC-CARE was more effective than having no intervention during the same period; however, this study could not determine its effectiveness compared to another active treatment. More research is needed to demonstrate the effectiveness of PC-CARE compared with longer evidence-based treatments using a randomized design. This research should also include an economic analysis of comparative costs and returns on investment.

Another limitation is that findings may not be generalizable to children not represented by the study population. While the sample was diverse from a racial/ethnic standpoint, it was skewed toward highly educated caregivers, primarily biological families, and caregivers with relatively low reported pre-intervention parenting stress. Furthermore, children did not need a mental health diagnosis or minimum symptom severity to receive services. Thus, results may not be generalizable to children with significant mental health concerns in a mental health treatment setting, though other studies of PC-CARE showed similar improvements for children in mental health treatment [11]. Future research should include RCTs within mental health treatment settings and examine PC-CARE’s effectiveness for families with varying backgrounds and languages.

The rates of participation of families in this study are a limitation, though they indicate promise and need for further study of engagement with and retention in PC-CARE. Compared to the 25% of children with externalizing behaviors who would be expected to receive mental health treatment [6], 48% of referred families participated in at least one appointment, and 39% completed post-intervention assessments. The 52% of families who did not participate in any appointments were a combination of families who never responded to attempted contacts or declined after the study was described. The specific reasons for not engaging with this study are unfortunately unknown, though the findings that 82% of families who attended a first appointment completed the study even when placed on the waitlist is encouraging. Future studies are necessary to examine factors related to families’ non-responsiveness to referral to PC-CARE or other mental health interventions in primary care clinical processes to better estimate true engagement and adherence.

## Clinical implications

Recent federal policies advocate the use of evidence-based treatments for mental health concerns, including children’s externalizing behaviors (e.g., Families First Prevention Services Ace-H.R. 5456, FFPSA). As brief interventions have been found to be effective and better able to address access and retention concerns [9], providers should seek to incorporate these interventions in their repertoires. While many more time intensive interventions are highly effective, they may not be necessary for all families. PC-CARE may be a helpful addition for providers in a variety of settings, including primary care, early intervention, schools, foster family agencies, and mental health agencies. Because it is brief and effective, incorporating PC-CARE may allow providers to see more families and have shorter waiting lists, while maintaining confidence that the services they provide are effective.

## Summary

There is a growing recognition of the need for brief, effective interventions for children’s externalizing problems which are more accessible and appealing to families and keep them engaged. The current study is the first RCT of PC-CARE, a dyadic intervention for children aged 2–10 years designed to be brief, accessible, and easily implemented in a variety of settings. Participants were 49 parent-child dyads randomly assigned to either PC-CARE treatment (N = 26) or waitlist control (N = 23). Results demonstrated an 81% retention rate for families participating in PC-CARE and indicated that participation in PC-CARE was associated with improvements in children’s externalizing problems and adaptive skills, reductions in parenting-related stress, and increases in parents’ use of positive communication skills compared to the waitlist control. The implications of this study suggest that PC-CARE is a brief and effective parenting intervention to improve externalizing behaviors in children and reduce parenting stress.
